# Quantitative phosphoproteomics reveals molecular pathway network in wheat resistance to stripe rust

**DOI:** 10.1007/s44154-024-00170-0

**Published:** 2024-07-01

**Authors:** Pengfei Gan, Chunlei Tang, Yi Lu, Chenrong Ren, Hojjatollah Rabbani Nasab, Xufeng Kun, Xiaodong Wang, Liangzhuang Li, Zhensheng Kang, Xiaojie Wang, Jianfeng Wang

**Affiliations:** 1 State Key Laboratory of Crop Stress Resistance and High-Efficiency Production, College of Plant Protection, Northwest A&F University, Yangling, 712100 Shaanxi China; 2 Plant Protection Station of Xinjiang Uygur Autonomous Region, Urumqi, 830049 Xinjiang China; 3 Plant Protection Research Department,Agricultural and Natural Resource Research and Education Center of Golestan, Agricultural Research,Education and Extension Organization (AREEO), Gorgan, Iran

**Keywords:** Wheat, *Puccinia striiformis* f. sp. *tritici*, Phosphoproteomics

## Abstract

Protein phosphorylation plays an important role in immune signaling transduction in plant resistance to pathogens. Wheat stripe rust, caused by *Puccinia striiformis* f. sp. *tritici* (*Pst*), severely devastates wheat production. Nonetheless, the molecular mechanism of wheat resistance to stripe rust remains limited. In this study, quantitative phosphoproteomics was employed to investigate the protein phosphorylation changes in wheat challenged by *Pst*. A total of 1537 and 2470 differentially accumulated phosphoproteins (DAPs) were identified from four early infection stage (6, 12, 18 and 24 h post-inoculation) in incompatible and compatible wheat-*Pst* interactions respectively. KEGG analysis revealed that Oxidative Phosphorylation, Phosphatidylinositol Signaling, and MAPK signaling processes are distinctively enriched in incompatible interaction, while Biosynthesis of secondary metabolites and RNA degradation process were significantly enriched in compatible interactions. In particular, abundant changes in phosphorylation levels of chloroplast proteins were identified, suggesting the regulatory role of photosynthesis in wheat-*Pst* interaction, which is further emphasized by protein-protein interaction (PPI) network analysis. Motif-x analysis identified [xxxxSPxxxx] motif, likely phosphorylation sites for defensive response-related kinases, and a new [xxxxSSxxxx] motif significantly enriched in incompatible interaction. The results shed light on the early phosphorylation events contributing to wheat resistance against *Pst*. Moreover, our study demonstrated that the phosphorylation levels of Nucleoside diphosphate kinase TaNAPK1 are upregulated at 12 hpi with CYR23 and at 24 hpi with CYR31. Transient silencing of *TaNAPK1* was able to attenuate wheat resistance to CYR23 and CYR31. Our study provides new insights into the mechanisms underlying *Pst*-wheat interactions and may provide database to find potential targets for the development of new resistant varieties.

**Supplementary Information:**

The online version contains supplementary material available at 10.1007/s44154-024-00170-0.

## Introduction

Stripe rust, caused by the obligate biotrophic fungus *Pst*, adversely impacts wheat production, which causes quality declines and substantial yield losses, making a significant menace to global food security (Chen et al. 2014). Planting resistant wheat cultivars is the most effective approach to control stripe rust. With the rapid evolution of new races and *Pst* virulence emerging, wheat varieties, however, lose their resistance usually in a short period (Zeng et al. 2022). Theres is therefore is a pressing need to better understand *Pst*-wheat interactions at a molecular basis and identify new molecular targets for more effective and durable resistance.

In nature, plants have evolved two layers of innate immune systems to counteract various pathogens’ invasion. Plant pattern recognition receptors (PRRs) located in the cytoplasmic membrane recognize pathogen-associated molecular patterns (PAMPs) to trigger the first layer immunity, called PAMP-triggered immunity (PTI). In turn, pathogen release effectors into plant cells to disrupt host immunity and enhance pathogen pathogenicity. When the pathogen effectors are recognized by the corresponding plant resistance (R) proteins directly or indirectly, the effector-triggered immunity (ETI) is activated (Jones and Dangl 2006; Yuan et al. 2021; Zhang et al. 2020). PTI and ETI occur rapidly, in which post-translational modifications (PTMs) within the proteome are essential for rapid cell reprogramming, defense signal transduction, cell homeostasis maintaining and so on (Liu et al. 2015). Protein phosphorylation, as an extensive type of protein modification, plays significant roles in regulating plant immunity (Park et al. 2012). The phosphorylation status of key regulatory proteins during plant-pathogen interactions influences the activation of immune signaling. During PTI, FLAGELLIN SENSING 2 (FLS2) can recognize the flg22 peptide derived from bacterial flagellin, interacts with BIK1 (BOTRYTIS-INDUCED KINASE 1). Upon pathogen infection, the perception of the PAMP flg22 induces the formation and phosphorylation of the FLS2/BAK1 (BRASSINOSTEROID INSENSITIVE 1-ASSOCIATED RECEPTOR KINASE1) complex. Activated BAK1 phosphorylates BIK1, leading to BIK1 dissociation from the FLS2/BAK1 complex. The released BIK1 then phosphorylates the N-terminus of NADPH oxidase RbohD, thereby triggering production of reactive oxygen species (ROS). Additionally, BIK1 phosphorylates and activates CNGC4, facilitating calcium influx (Zhang et al. 2023).

The modulation of phosphorylation modifications by pathogen effectors during the plant ETI response is a mechanism that contributes to pathogenicity. *Pseudomonas*. *syringae* effectors AvrRpm1 and AvrB rely on the plant kinase RPM1-INDUCED PROTEIN KINASE (RIPK) to regulate the phosphorylation of the positive immune regulator RPM1-INTERACTING PROTEIN 4 (RIN4) (Lee et al. 2015). To gain a deeper understanding of the phosphorylation events associated with ETI, a phosphoproteomic screening in *Arabidopsis thaliana* identified 109 phosphorylated residues on membrane-associated proteins during the activation of the intracellular RPS2 receptor in response to avrRpt2 (Kadota et al. 2018). In rice infected with *Xanthomonas oryzae* pv. *oryzae* (*Xoo*), 762 differentially phosphorylated proteins were identified, including transcription factors, kinases, epigenetic regulating factors, and disease-resistant proteins, suggesting their potential involvement in *Xoo* resistance (Hou et al. 2015). During tomato-*Pseudomonas syringae* infection, 79 phosphopeptides exhibited differential accumulation in tomato leaves, indicating dynamic changes in protein and phosphoprotein levels (Yu et al. 2021). Phosphorylation modifications also mediate cross-talk between PTI and ETI signaling pathways. MAPK cascade is an important phosphorylation signaling system shared by both PTI and ETI. It consists of three protein kinases, namely MAPKKK, MAPKK, and MAPK. MAPKKK activates MAPKK through phosphorylation, and then MAPKK activates MAPK. Enhancing the participation of MAPK cascade in immune responses can induce robust immune reactions and eliminate various pathogens (Yamamizo et al. 2006).

In eukaryotes, approximately one-third of proteins undergo phosphorylation modifications, highlighting the ubiquity and significance of this process (Sefton 2001). Phosphoproteomics provides a comprehensive approach to understand host-pathogen interactions from a global perspective (Olsen et al. 2006). In recent years, advances in phosphor-proteomics have provided powerful tools for investigating signal transduction pathways and identifying various types of PTMs that are involved in regulating protein functions (Luan 2003; Barman and Ray 2020).

As an obligate biotrophic fungus, *Pst* depends entirely on its host for growth and reproduction. The urediniospores of the fungus produce germ tubes around 3 hours after landing on the wheat leaf surface and then the germ tubes invade through the leaf stomata (Wang et al. 2007; Kang et al. 2002). At 8–12 hpi, a substomatal vesicle is formed in the stomatal cavity, and over the course of 18–24 hpi, a primary infection hypha and haustorial mother cell (HMC) are formed (Chen et al. 2014; Kadota et al. 2018; Zhang et al. 2011). After penetration of plant cell wall, the haustorial mother cell produces a root-like structure called the haustorium, which is specialized not only for extracting nutrients from the host but also for secreting small proteins (Cantu et al. 2013; Garnica et al. 2014; Hovmøller et al. 2011). These secreted proteins include effectors that play a key role in establishing infection (Lorrain et al. 2019). In this study, we performed quantitative phosphoproteomics analysis to identify the changes in phosphorylation level in the early infection stage of *Pst* to analyze the molecular events that contribute to stripe rust resistance in wheat. In addition to the common biological pathways identified in both compatible and incompatible wheat-*Pst* interaction, interestingly distinctive biological pathways were enriched in incompatible interaction, including Oxidative Phosphorylation, Phosphatidylinositol Signaling, MAPK signaling processes and so on, which likely contribute to wheat stripe rust resistance. And in compatible interactions, Biosynthesis of secondary metabolites and RNA degradation process were significantly enriched. Our results provide new insights into the molecular mechanisms underlying *Pst*-wheat interactions and may reveal potential targets for the development of new resistant varieties.

## Results

### Identification and quantification of phosphorylated proteins in *Pst*-infected wheat

**Fig. 1:**
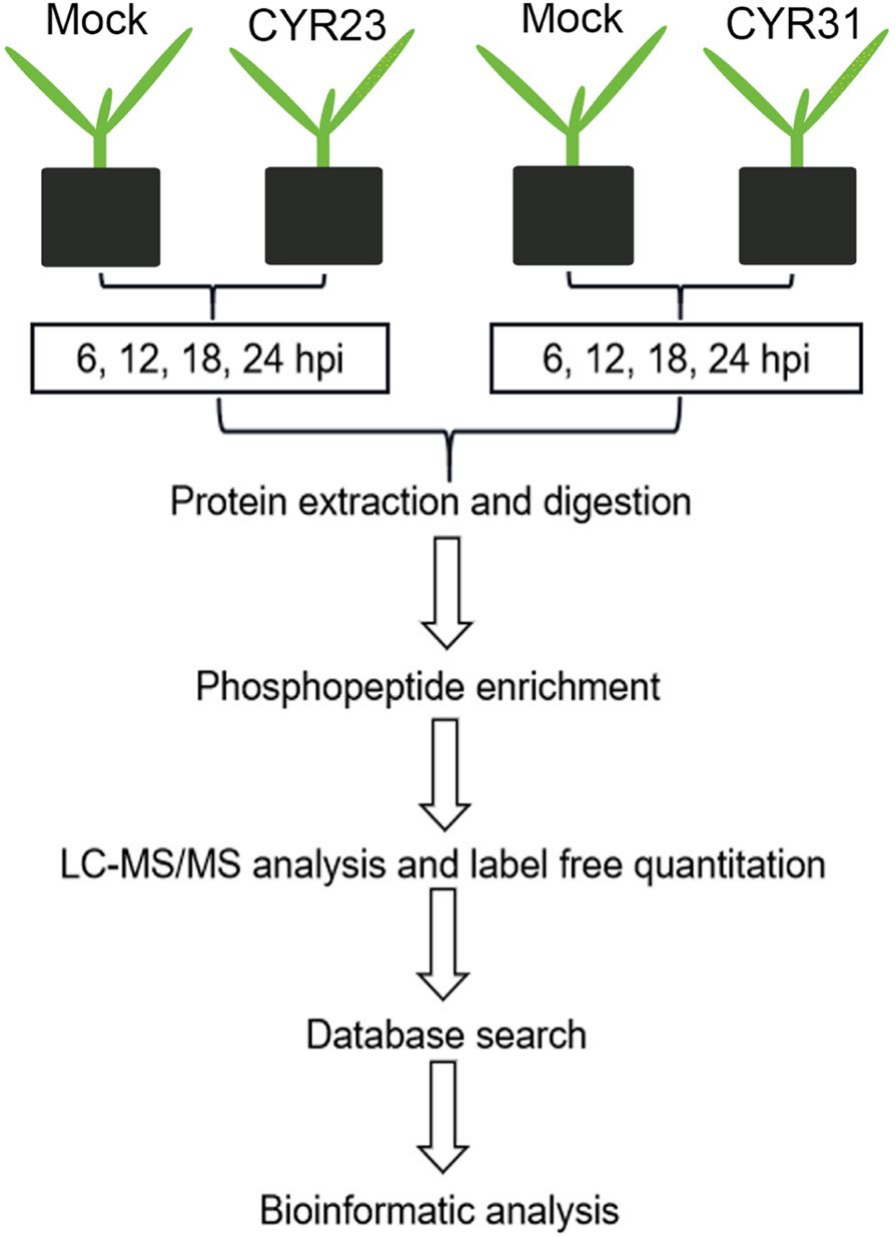
Work flow for the phosphoproteomics strategy for quantifying the phosphorylation changes in the interaction between wheat and *Pst*. MOCK is a non-inoculated control, CYR23 means inoculated with *Pst* race CYR23, and CYR31 means inoculated with *Pst* race CYR31

The phosphorylation modifications occurring during the interaction between wheat and *Pst* were investigated through a label-free quantitative phosphoproteomics approach. The materials used in the experiment were Fielder at the two-leaf stage. The *Pst* race CYR23 and *Pst* race CYR31 were separately inoculated. Their respective control groups were inoculated with sterilized water. Following inoculation, leaf samples were collected at four time points: 6, 12, 18, and 24 hours, for total protein extraction. The extracted total proteins underwent trypsin digestion, phosphopeptide enrichment, and LC-MS/MS analysis. The resulting data were utilized for subsequent analysis (Fig. 1).

**Fig. 2:**
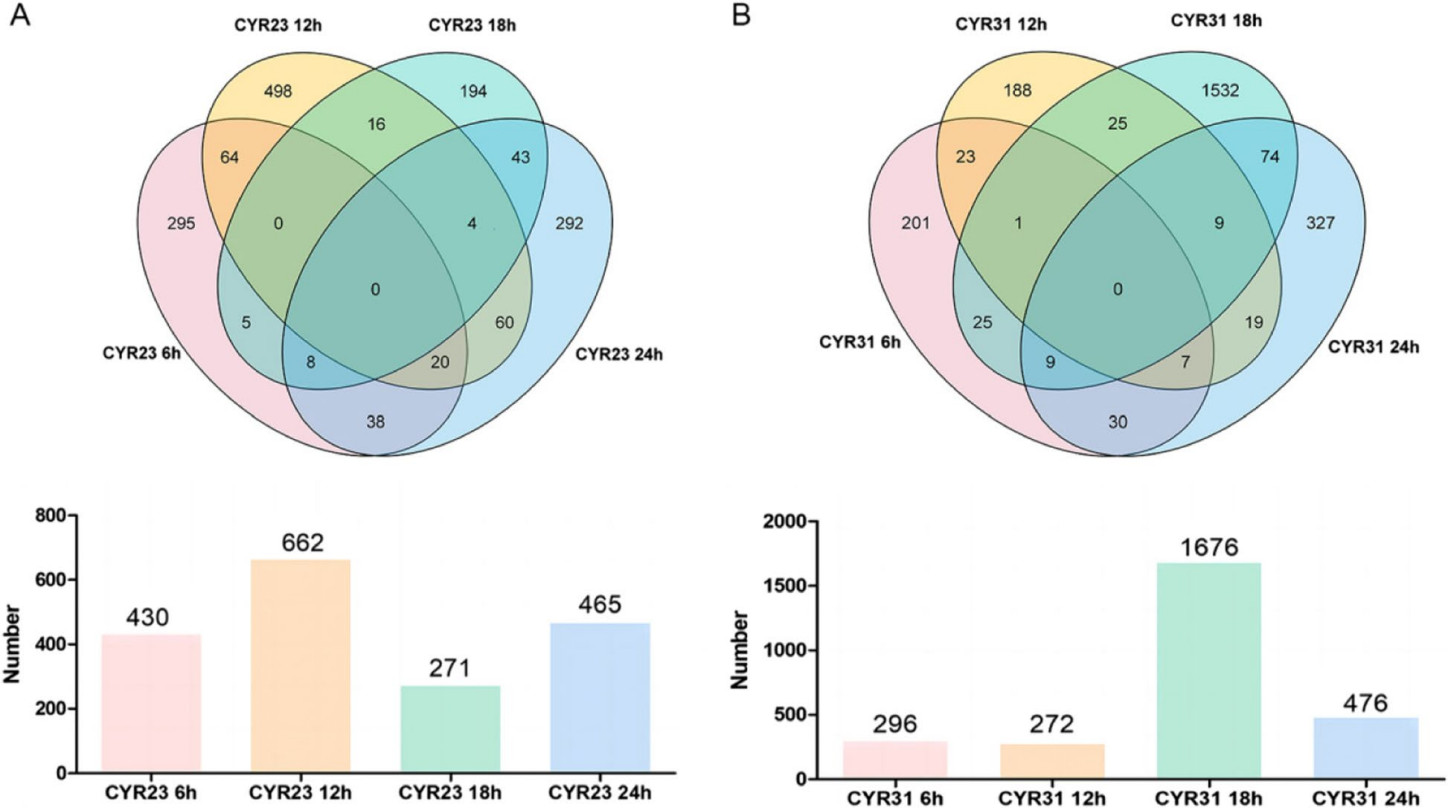
The statistical results of DAPs identified in wheat-*Pst* interaction. The Venn diagrams and bar charts display the overlaps and quantities of DAPs identified at four different time points (6 h, 12 h, 18 h, and 24 h) after inoculation with *Pst* race CYR23 (**A**) and CYR31 (**B**)

In the incompatible interaction combination, the mock group detected 1765, 2913, 2927, and 2242 peptide segments at the 6, 12, 18, and 24 hpi, respectively. In the samples inoculated with CYR23, 6067, 1555, 2378, and 2077 peptide segments were detected at the four time points. In the compatible interaction combination, the mock group detected 2464, 2042, 1409, and 3415 peptide segments at the 6, 12, 18, and 24 hpi, respectively. In the samples inoculated with CYR31, 2235, 2339, 3547, and 2488 peptide segments were detected at the four time points (Table S1). A total of 18,012 and 18,662 phosphorylated peptides were obtained in incompatible and compatible interaction by comparing to the MOCK. And 1537 differentially accumulated proteins (DAPs) with a fold change above 2 or below 0.5, and a *P*-value less than 0.05 were identified in *Pst* race CYR23 inoculated wheat plants, including 430, 662, 271 and 465 DAPs at 6, 12, 18 and 24 hpi respectively (Fig. 2A, Table S1). And 2470 DAPs were identified in *Pst* race CYR31 infected wheat plants, with 296, 272, 1676 and 476 DAPs at 6, 12, 18, and 24 hpi respectively (Fig. 2B, Table S1).

### KEGG annotation of the DAPs

**Fig. 3:**
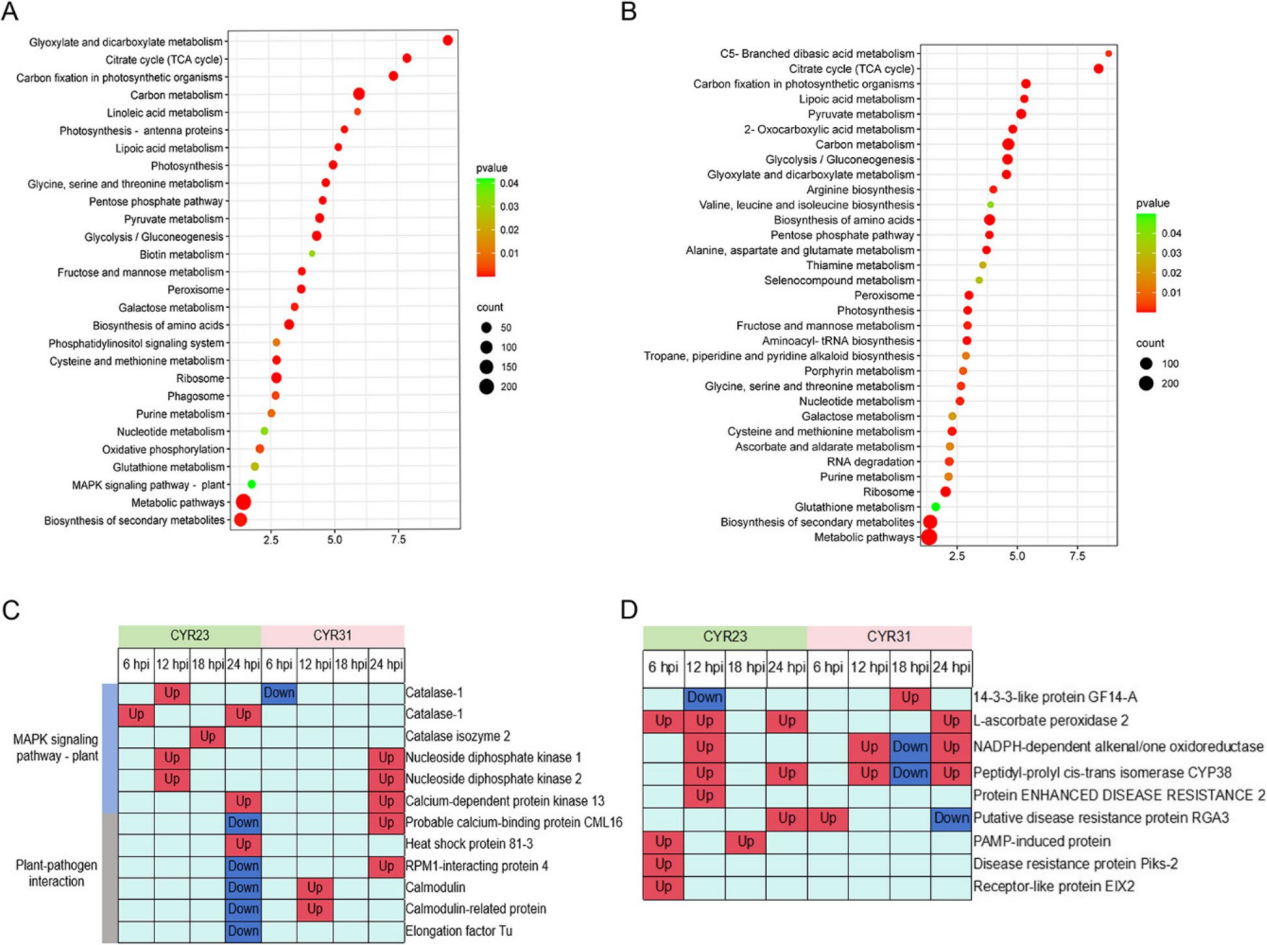
KEGG analyses of the total DAPs at four time points during infection of *Pst* race CYR23 (**A**) and CYR31 (**B**). **C** The heat map shows the phosphorylation level changes of proteins related to the MAPK signaling pathway enriched by total DAPs in incompatible and compatible interactions, as well as the plant-pathogen interaction pathway enriched at 18 h. **D** The phosphorylation level changes of resistant -related proteins in incompatible and compatible interactions at different time points. Up represents an upregulation in phosphorylation level, while Down represents a downregulation in phosphorylation level

KEGG functional clustering enrichment was conducted to determine the relevant pathways alongside the DAPs identified in wheat inoculated with CYR23 and CYR31, respectively. 399 DAPs in wheat-*Pst* CYR23 interaction were enriched in 28 KEGG terms, and 471 DAPs in wheat-*Pst* CYR31 interaction were classed into 33 KEGG terms. Twenty-one KEGG terms were enriched with DAPs related to both *Pst* race CYR23 and CYR31. These terms include Carbon metabolism, Glyoxylate and dicarboxylate metabolism, Carbon fixation in photosynthetic organisms, Citrate cycle (TCA cycle), Metabolic pathways, Glycolysis/Gluconeogenesis, Biosynthesis of amino acids, Ribosome, Photosynthesis, Glycine, serine and threonine metabolism, Peroxisome, Pentose phosphate pathway, Cysteine and methionine metabolism, Biosynthesis of secondary metabolites, Nucleotide metabolism, Pyruvate metabolism, Lipoic acid metabolism, Fructose and mannose metabolism, Galactose metabolism, Purine metabolism, and Glutathione metabolism (Fig. 3, Table S2). These pathways primarily participate in processes such as Carbohydrate metabolism, Energy metabolism, Amino acid metabolism, Metabolism of cofactors and vitamins, Transcription, Transport and catabolism. Lipid metabolism and Signal transduction as the phosphatidylinositol signaling system and the MAPK signaling pathway-plant pathways were specifically enriched in incompatible interaction (Fig. 3A), and Biosynthesis of secondary metabolites and RNA degradation were significantly enriched in compatible interaction (Fig. 3B). The KEGG clustering results of DAPs at each time point are illustrated in Fig. S1. In the incompatible interaction, the Carbon metabolism pathway was dominant at all the four stages. The most abundant signaling pathways were seen at 12 h, which included oxidative phosphorylation, pyruvate metabolism, photosynthesis, and other pathways related to plant immunity. At 18 h, the enriched pathways were those involved in metabolism, peroxisome, and others. At 24 h, the plant-pathogen interaction pathway was enriched. In the compatible interaction, at 6 h, the enriched pathways were Photosynthesis, Peroxisome, and Biosynthesis of amino acids. At 12 h, the dominant pathways were Peroxisome, Biosynthesis of amino acids, and Phosphatidylinositol signaling system. At 18 h, the enriched pathways were those of the ribosome and oxidative phosphorylation. At 24 h, the enriched pathways were those of Pyruvate metabolism, Sphingolipid metabolism, and Photosynthesis. By analyzing the major proteins involved in the MAPK signaling pathway-plant and the plant-pathogen interaction pathway (Fig. 3C), Two catalase-1 were significantly enriched in incompatible interactions, with upregulation in phosphorylation levels at 12 h and 6 h, and downregulation and no change in compatible interactions. The phosphorylation level of L-ascorbate peroxidase 2 was upregulated at 6, 12, and 24 h in incompatible interactions, and upregulated at 24 h in compatible interactions. Both catalase and L-ascorbate peroxidase are enzymes that clear H_2_O_2_, and their phosphorylation levels may be related to the ROS burst of wheat-*Pst* compatible interactions. EIX is related to the recognition of elicitor by receptor-like proteins, and its phosphorylation level is upregulated at 12 h in incompatible interactions. Other disease resistance-related proteins, such as Protein EDR2 (ENHANCED DISEASE RESISTANCE 2), Putative disease resistance protein RGA3, PAMP-induced protein, Disease resistance protein Piks-2, are all upregulated in phosphorylation levels during incompatible interactions(Fig. 3D).

### Features of the phosphoproteins and motif enrichment

**Fig. 4:**
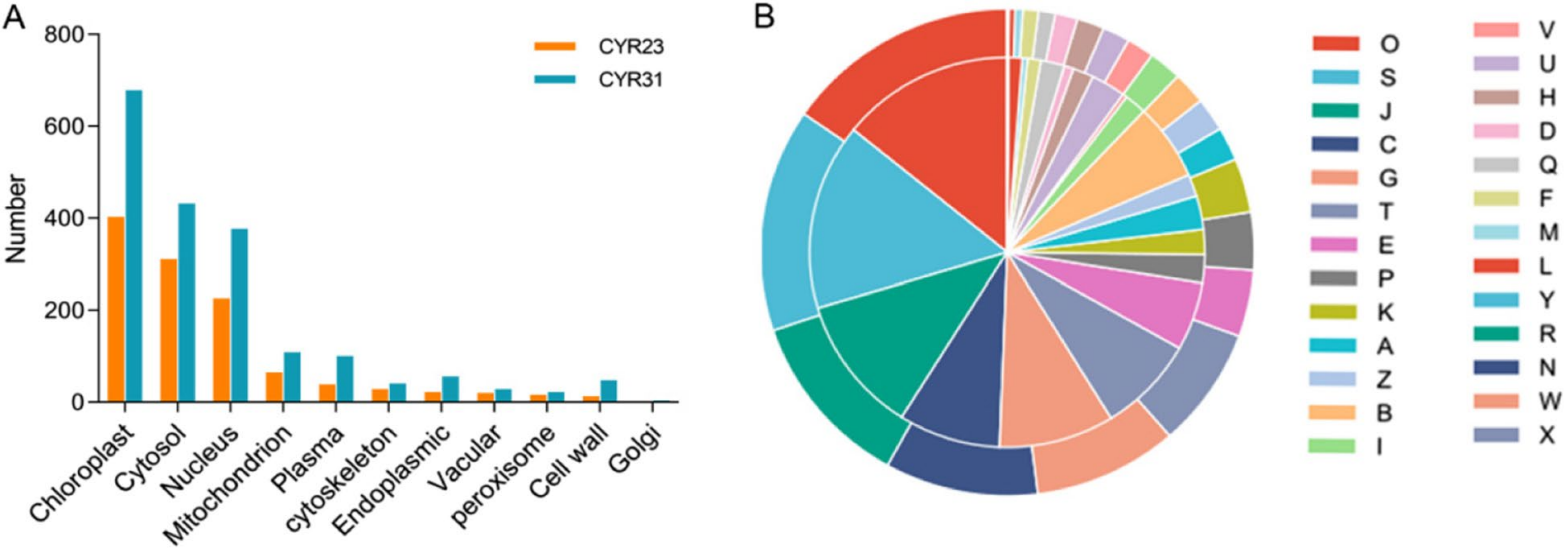
**A** Subcellular localization determined by CropGF for CYR23- and CYR31-related DAPs. **B** Double layer pie chart of eggNOG functional annotations represented in different colors for CYR23-related (Outer layer) and CYR31-related (Inner layer) DAPs. **A-**RNA processing and modification, **B-**Chromatin structure and dynamics, **C**-Energy production and conversion, **D**-Cell cycle control, cell division-chromosome partitioning, **E**-Amino acid transport and metabolism, **F**-Nucleotide transport and metabolism, **G**-Carbohydrate transport and metabolism, **H**-Coenzyme transport and metabolism, **I**-Lipid transport and metabolism, **J**-Translation, ribosomal structure and biogenesis, **K**-Transcription, **L**-Replication, recombination and repair, **M**-Cell wall/membrane/envelope biogenesis, **N**-Cell motility, **O**-Posttranslational modification, protein turnover, chaperones, **P**-Inorganic ion transport and metabolism, **Q**-Secondary metabolites biosynthesis, transport and catabolism, **R**-General function prediction only, **S**-Function unknown, **T**-Signal transduction mechanisms, **U**-Intracellular trafficking, secretion, and vesicular transport, **V**-Defense mechanisms, **W**-Extracellular structures, **X**-Mobilome: prophages, transposons, **Y**-Nuclear structure, **Z**-Cytoskeleton

CropGF (https://bis.zju.edu.cn/cropgf) (Xu et al. 2023) was used to predict the subcellular localization of DAPs. It was found that CYR23 and CYR31-related DAPs were mainly enriched in chloroplasts, followed by that in the cytosol and nucleus (Fig. 4A, Table S3). COG analysis performed with eggNOG revealed that the CYR23 and CYR31-related DAPs were involved in post-translational modification, protein turnover, chaperones; translation, ribosomal structure and biogenesis, energy production and conversion; carbohydrate transport and metabolism; signal transduction mechanisms. CYR23-related DAPs show a high proportion of involvement in inorganic ion transport and metabolism and defense mechanisms. And a high proportion of CYR31-related DAPs are associated with chromatin structure and dynamics (Fig. 4B, Table S4).

**Fig. 5:**
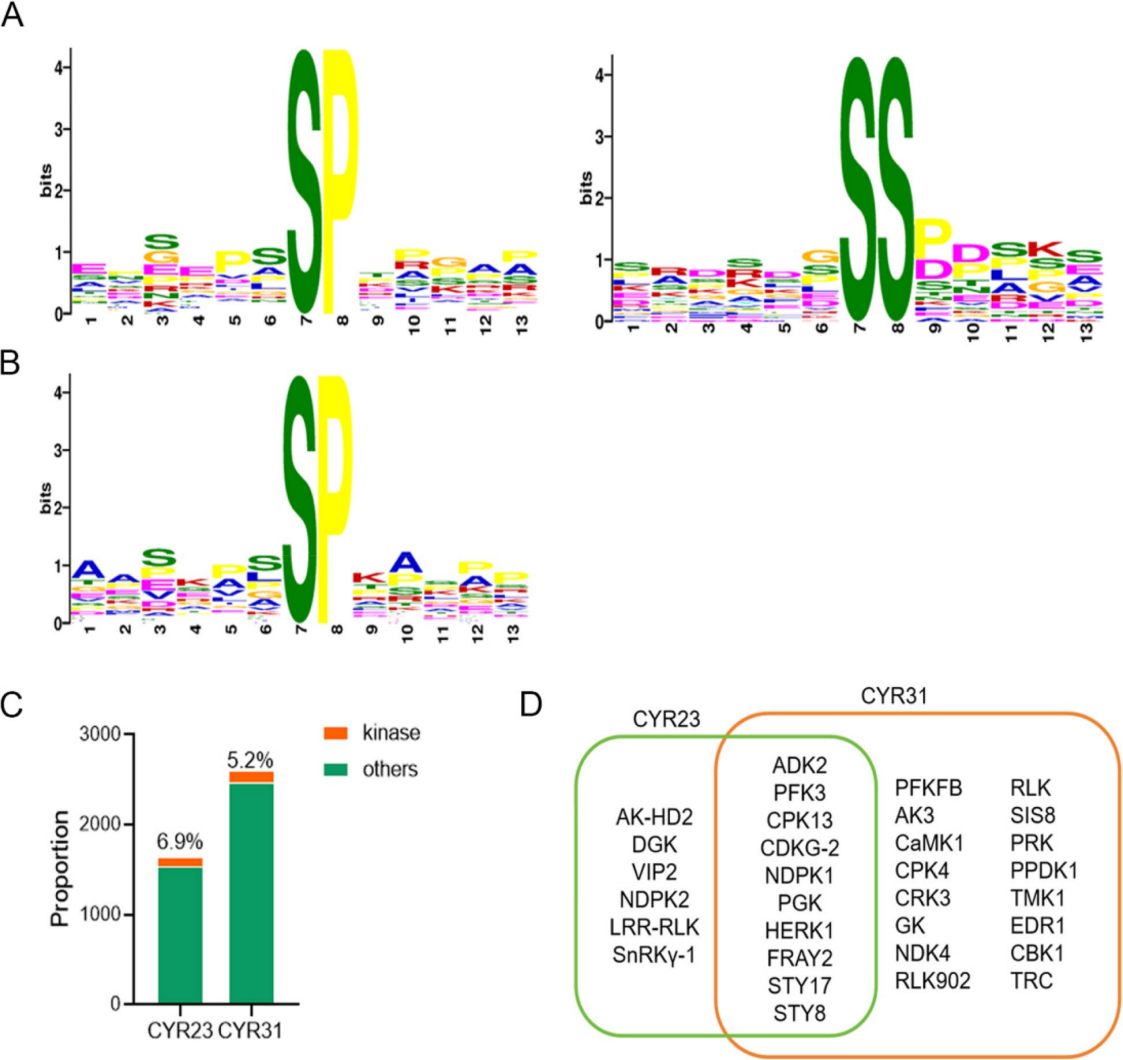
Motif-x enrichment analysis of both the CYR23-related (**A**) and CYR31-related (**B**) phosphoproteins with the MOMO tool returned three overrepresented motifs. **C** The bar chart displays the proportion of kinases among the DAPs in incompatible and compatible interactions. **D** The Venn diagram illustrates the comparison between CYR23 and CYR31, showing the shared and different kinase categories

The specificity of kinases for substrates is primarily determined by motifs that encompass phosphorylation sites. Thus, analyzing conserved motifs in differentially phosphorylated peptide segments can offer valuable insights into the identification of the kinases responsible for their phosphorylation. Analyses of upregulated phosphorylated peptide segments from CYR23 and CYR31 infected wheat plants utilizing the online tool Motif-X with S and T as the central residues within the phosphopeptide uncovered the presence of the serine-directed motif [xxxxSPxxxx] (Fig. 5A, B). Proteins harboring the [xxxxSPxxxx] motif have been reported as substrates for various kinase (van Wijk et al., 2014). Additionally, in incompatible interaction, [xxxxSSxxxx] motif were identified in phosphorylated peptide segments (Fig. 5A), which represent a novel kinase motif. The potential upstream kinases responsible for phosphorylation of this motif remain unclear.

Analysis of the quantity and types of differentially phosphorylated kinases identified in the CYR23 and CYR31 experimental groups revealed that there are a total of 106 kinases in the DAPs of CYR23, accounting for 6.9% of all CYR23-related DAPs. In the DAPs of CYR31, there were a total of 128 kinases, accounting for 5.2% of all CYR31-related DAPs (Fig. 5C). Among these kinases, six types were unique to CYR23-related DAPs, which included Bifunctional aspartokinase/homoserine dehydrogenase 2 (AK-HD2), D-glycerate 3-kinase (DGK), Inositol hexakisphosphate and diphosphoinositol-pentakisphosphate kinase VIP2 (VIP2), Nucleoside diphosphate kinase 2 (NDPK2), Probable LRR receptor-like serine/threonine-protein kinase (LRR-RLK), and SNF1-related protein kinase regulatory subunit gamma-1 (SnRKγ-1).In the case of CYR31-related DAPs, there were 16 types of kinases that were unique to this group, including 2-phosphoglycerate kinase (PGK), 6-phosphofructo-2-kinase/fructose-2,6-bisphosphatase (PFKFB), Adenylate kinase 3 (AK3), Calcium/Calmodulin-dependent serine/threonine-protein kinase 1 (CaMK1), Calcium-dependent protein kinase 4 (CPK4), CDPK-related kinase 3 (CRK3), Glycerol kinase (GK), Nucleoside diphosphate kinase 4 (NDPK4), Nucleoside diphosphate kinase IV (NDPK IV), Probable inactive receptor kinase RLK902 (RLK902), Probable receptor-like protein kinase (RLK), Probable serine/threonine-protein kinase SIS8 (SIS8), Protein-ribulosamine 3-kinase (PRK), Pyruvate, phosphate dikinase 1 (PPDK1), Receptor protein kinase TMK1 (TMK1), Serine/threonine-protein kinase EDR1 (EDR1), Serine/threonine-protein kinase CBK1 (CBK1), and Serine/threonine-protein kinase tricorner (TRC).The kinases that were common to both CYR23 and CYR31-related DAPs included Adenosine kinase 2 (ADK2), ATP-dependent 6-phosphofructokinase 3 (PFK3), Calcium-dependent protein kinase 13 (CPK13), Cyclin-dependent kinase G-2 (CDKG-2), Nucleoside diphosphate kinase 1 (NDPK1), Receptor-like protein kinase HERK 1 (HERK1), Serine/threonine-protein kinase fray2 (FRAY2), Serine/threonine-protein kinase STY17 (STY17), and Serine/threonine-protein kinase STY8 (STY8) (Fig. 5D).

### Regulatory network contributing to *Pst* resistance

**Fig. 6:**
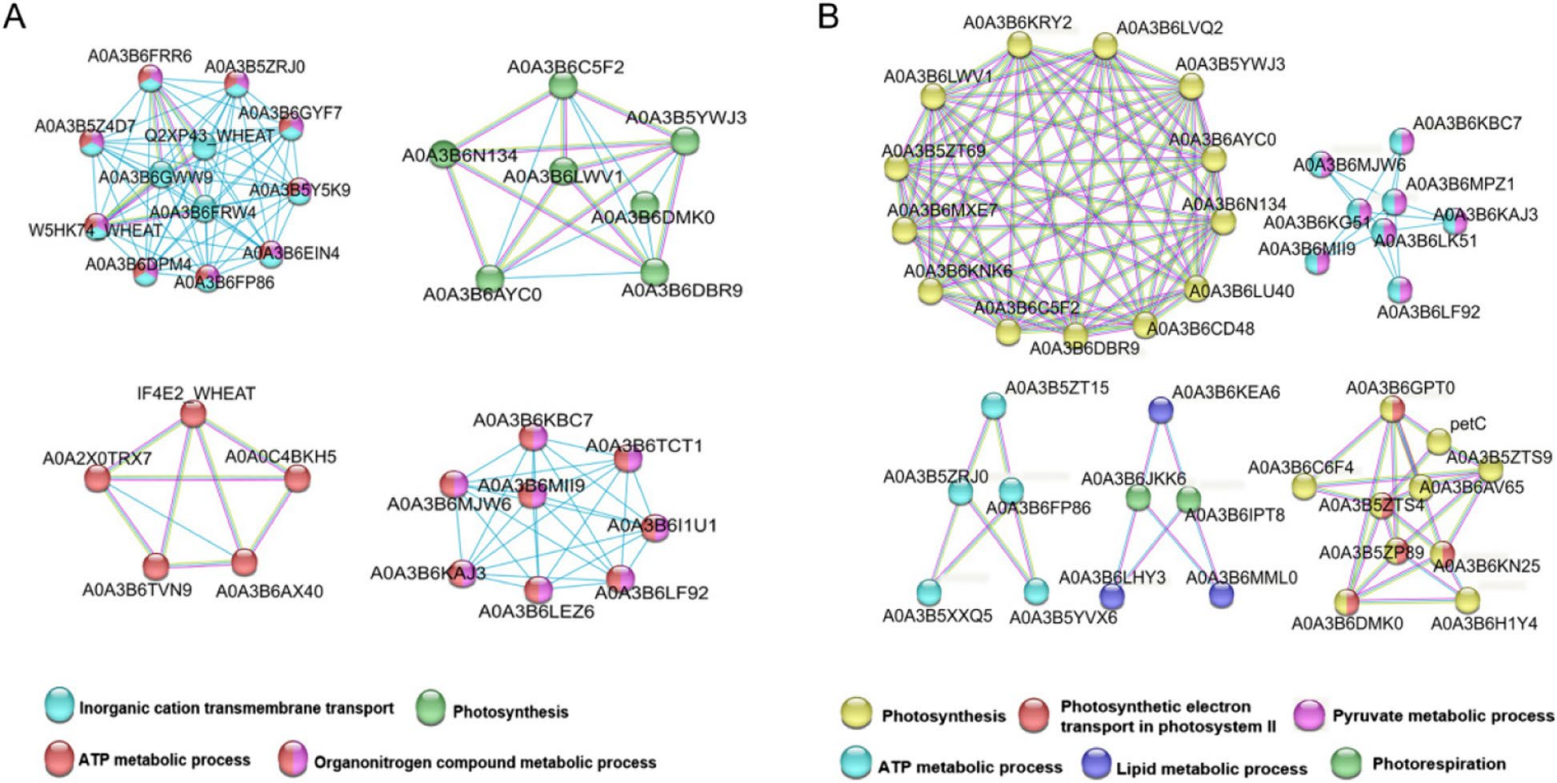
Protein−protein networks determined by the STRING environment contrasting the DRP sequences against the *Triticum aestivum* databank. The CYR23 (**A**) and CYR31 (**B**) -related DAPs. Different colored balls represent different biological processes

By utilizing String 12.0, with confidence score threshold set to 0.7, PPI networks were constructed for the cumulative DAPs. For CYR23-related phosphoproteins, we found statistically significant enrichment of the biological processes of Inorganic cation transmembrane transport, Photosynthesis, ATP metabolic process and Organonitrogen compound metabolic process (Fig. 6A). In the CYR31-related group, the phosphoproteins were enriched in Photosynthesis, Photosynthetic electron transport in photosystem II, Pyruvate metabolic process, Lipid metabolic process, ATP metabolic process and Photorespiration (Fig. 6B). Table S1 summarizes the annotation of genes involved in the PPI networks and their corresponding gene IDs in the IWGSCv1.1 version.

### Functional verification of TaNDPK1 in wheat resistance to *Pst*

**Fig. 7:**
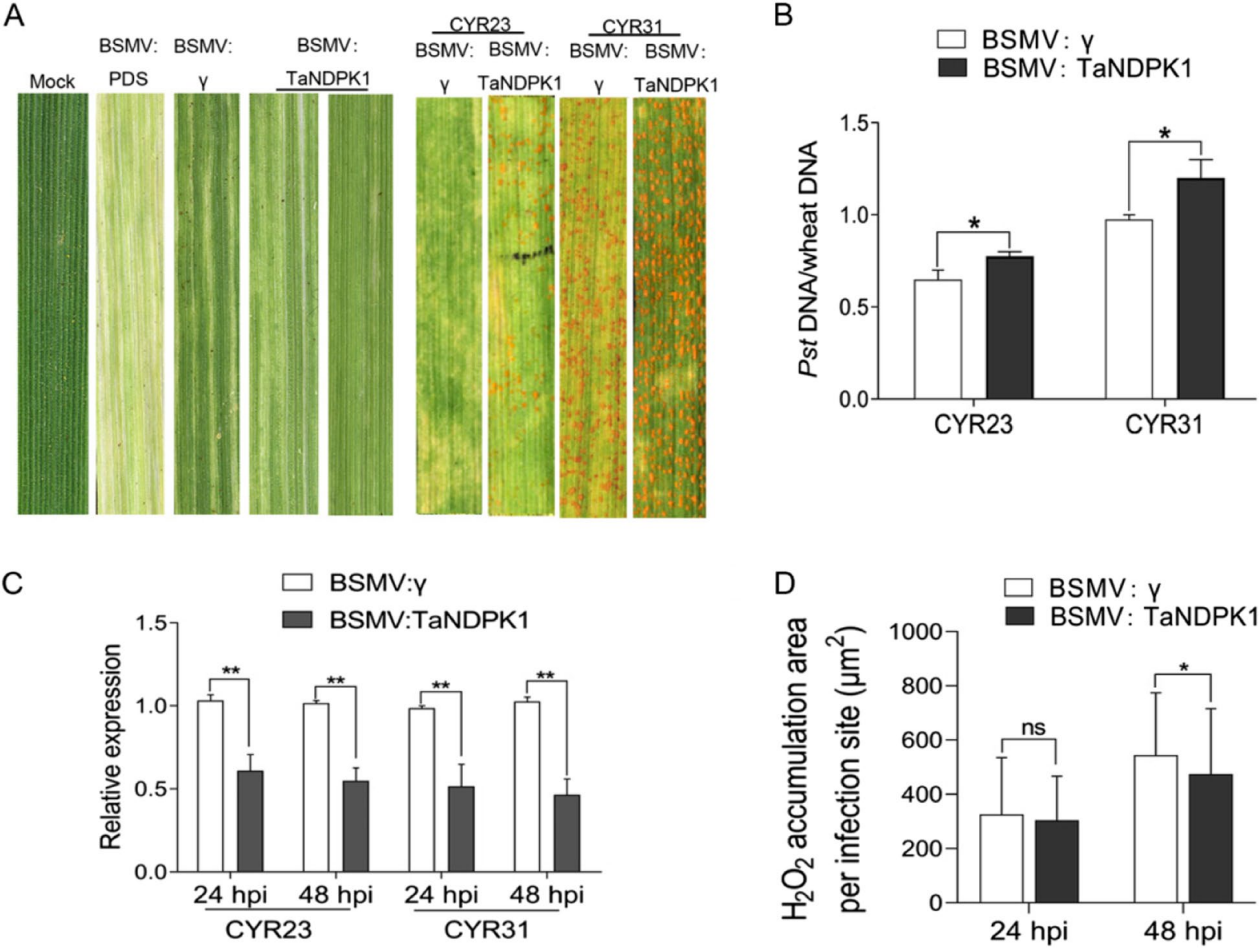
Functional verification of *TaNDPK1* in wheat resistance to *Pst*. **A** BSMV-mediated gene silencing of *TaNDPK1*, photos left show the phenotype of virus infected plants, right panel show the phenotype of wheat inoculated with CYR23 and CYR31, photos taken at 14 dpi. **B** Relative biomass of *Pst* conduceted by qRT-PCR, *PstEF* and *TaEF* were used as endogenous reference genes. The mean values ± SD were calculated from three biological replicates. Statistical significance was analyzed by an unpaired two-tailed Student’s t test, *: *P* < 0.05. **C** Silencing efficiency of *TaNDPK1* in BSMV-infected wheat after *Pst* inoculation. The mean values ± SD were calculated from three from three biological replicates. *: *P* < 0.05, **: *P* < 0.01. **D** H_2_O_2_ area in the infection sites inoculated CYR23. H_2_O_2_ were stained by DAB and observed using microscope, the areas were measured using Cellsens software. The mean values ± SD were calculated from three from thirty infection sites. Statistical significance was analyzed by an unpaired two-tailed Student’s t test. *: *P* < 0.05, ns: no significant difference

Nucleoside diphosphate kinase (NDPK) is a widely distributed enzyme that catalyzes the reversible transfer of γ-phosphate from triphosphonucleosides to diphosphonucleosides, and plays a role in maintaining the pool of ribonucleotides and deoxyribonucleotides in cells. It is also known to have important functions in host-pathogen interactions (Kapoor and Varshney 2020). In this study, the phosphorylation level of NDPK1 was upregulated in the incompatible interaction at 12 h and compatible interaction at 24 h, to verify the function of NDPK1 in the interaction between wheat and *Pst*. VIGS experiments were conducted to transiently silence *TaNDPK1* in Fielder, and the plants were then inoculated with CYR23 and CYR31. After 14 dpi, the spore production on the inoculated leaves was observed. The results showed that in the experiment with CYR23 inoculation, the *TaNDPK1* silenced plants had obvious spore production on the leaves, while the control group did not. In the experiment with CYR31 inoculation, the number of spores on the *TaNDPK1* silenced plants was significantly higher than on the control group (BSMV:γ) leaves (Fig. 7A). Compared to the control group, *TaNDPK1*-silenced plants showed a significant increase in biomass (Fig. 7B). qRT-PCR analysis confirmed efficient silencing of the *TaNDPK1* gene in *TaNDPK1*-silenced plants, as evidenced by a significant decrease in transcript levels compared to the control group (BSMV:γ) (Fig. 7C). Furthermore, the accumulation of H_2_O_2_ induced by CYR23 was significantly reduced in *TaNDPK1*-silenced plants (Fig. 7D). These findings indicate that *TaNDPK1* positively regulates wheat resistance to *Pst*.

## Discussion

This study employed label-free quantitative proteomics technology based on mass spectrometry to explore the phosphoproteome and molecular mechanisms involved in wheat resistance against stripe rust caused by *Pst*. The results reveal the phosphorylation events in the early wheat-*Pst* interaction stage, providing important molecular clues for uncovering the resistance mechanism.

The presence of exclusive DAPs in wheat plants challenged by *Pst* race CYR23 and CYR31 indicates distinct molecular responses triggered by these two races in wheat, which may contribute to wheat immunity in the incompatible interaction, and the survival of the obligate biotrophic rust fungal in susceptible wheat plants. The presence of a substantial number of proteins located in chloroplasts suggests their crucial role in plant immunity, adding to the comprehensive understanding of defense mechanisms in plants. Chloroplasts, plant-specific organelles responsible for photosynthesis, significantly regulate plant responses to biotic stress (Medina-Puche et al. 2020; Zhao et al. 2016). They play a crucial role in plant immune responses by perceiving danger signals, serving as a source of Ca^2+^ and ROS signals, transmitting the signals to the nucleus, leading to the expression of defense-related genes, including those involved in salicylic acid synthesis, and activating PTI in response to PAMPs (Chan et al. 2016; Nomura et al. 2012; Serrano et al. 2016). In the interaction between plants and pathogenic fungi, there are two peaks of ROS production (Castro et al., 2021). The first non-specific phase of ROS production is associated with respiratory burst oxidase homolog (RBOH) activity occurring at the cell membrane, while the second peak of ROS production is associated with ETI specificity and occurs in the chloroplast (Shapiguzov et al., 2012). RBOHD is a membrane-localized protein with six conserved transmembrane helices and cytoplasmic N and C termini. Upon perception of PAMPs by PRRs, calcium influx mediated by the Arabidopsis CPK5, a calcium-dependent protein kinase, leads to phosphorylation of RBOHD and various intracellular protein kinases induce phosphorylation of the N-terminal activation domain of RBOHD, promoting ROS production (Dubiella et al. 2013). ETI is often accompanied by a hypersensitive response (HR), a type of programmed cell death (PCD) in plants (Laflamme et al. 2020). During HR, chloroplasts are the main source of H_2_O_2_, which acts as a defense signaling molecule and induces nuclear gene expression (Yao and Greenberg 2006). The stromules can facilitate the direct transport of H_2_O_2_ produced in chloroplasts to the nucleus (Brunkard et al. 2015). Upon transportation to chloroplasts, the *Pst* effector protein *Pst*_12806 from *Pst* interacts with the subunit TaISP (Iron-Sulfur protein) of plant cytochrome cb6f protein, leading to disruption of plant photosynthesis, inhibition of chloroplast-mediated ROS production, and facilitation of pathogen colonization within the plant (Xu et al. 2019). By hijacking the cytoplasmic cytochrome b6/f complex, the *Pst* effector proteins *Pst*_4 and *Pst*_5 impede its transport to the chloroplasts, resulting in the inhibition of chloroplast ROS production and the promotion of stripe rust colonization (Wang et al. 2021). *Pst* infection leads to an enrichment of phosphorylated chloroplast proteins, signifying the potential role of chloroplasts as a central sensing hub in mediating plant immune responses. Moreover, chloroplast proteins have significant potential in striking a balance between crop yield and disease resistance by efficiently allocating energy between plant growth and defense mechanisms (Chen et al. 2023; Medina-Puche et al. 2020).

The KEGG analysis of DAPs identified in both *Pst* races showed enrichment in various metabolism-related pathways, such as carbohydrate metabolism, energy metabolism, and amino acid metabolism. This suggests the importance of metabolic reprogramming in the defense response against *Pst*. Additionally, the significant enrichment of DAPs related to CYR23 in the phosphatidylinositol signaling system and MAPK signaling pathway-plant further highlights the involvement of these pathways in *Pst* resistance. The important regulatory role of plant MAPK cascade signaling in plant defense against diseases has been recognized for a long time. The core members MPK3/MPK6/MPK4 regulate multiple disease-related processes, including ethylene synthesis, phytoalexin synthesis, secondary metabolite synthesis with antimicrobial activity, stomatal immunity, HR response, and expression of disease resistance genes (Zhang et al. 2018). The proteins that we enriched in this pathway mainly include NDPKs (Nucleoside diphosphate kinases), CaM (Calmodulin)/CML (Calmodulin-like), and Cat (catalase) (Moon et al. 2003). NDPKs enhance the phosphorylation of H_2_O_2_-activated MPK3/6. The instantaneous and rapid increase in cytosolic calcium concentration Ca^2+^ is one of the necessary early cellular responses in plant immunity. Once the host identifies the elicitors generated by pathogens, it triggers the activation of the signal transduction system, leading to the release of Ca^2+^. The free Ca^2+^ concentration in the extracellular solution is much higher than that in the resting cytoplasm (Demidchik et al. 2018; Luan and Chao 2021). The influx of Ca^2+^ into the cytoplasm can stimulate the activation of calcium-binding proteins such as CaMs and CMLs, subsequently triggering the synthesis of nitric oxide (NO) and initiating a primary immune response, which includes the HR. The downregulation of CaM phosphorylation levels in incompatible and compatible interactions suggests that important Ca^2+^ sensor categories are tightly regulated in their activity. Furthermore, NO may synergistically regulate HR and PCD in conjunction with ROS (Ma et al. 2008). In plants, catalase (CAT) primarily functions to scavenge hydrogen peroxide (H_2_O_2_) from ROS, thereby maintaining a stable ROS level (Sofo et al. 2015).Additionally, CAT acts upstream of autophagy in RMP1-mediated HR-PCD (Sertsuvalkul et al. 2022). In addition, the 14–3-3 protein acts as a scaffold and activator to regulate RLCK phosphorylation of MAPKKK5, promote MAPK activation, and enhance plant resistance (Dong et al. 2023; Lozano-Durán 2015). The phosphatidylinositol signaling system plays a crucial role in transmitting immune defense signals in plants (Abd-El-Haliem and Joosten 2017; Lozano-Durán 2015). Inositol hexakisphosphate and diphosphoinositol-pentakisphosphate kinase VIP2 are significantly regulated. In Arabidopsis, the homologous gene *VIH2* of VIP1 plays a role in regulating jasmonate perception and plant defense against herbivorous insects and necrotrophic fungi (Laha et al. 2015). Phosphatidylinositol 3,4,5-trisphosphate 3-phosphatase and protein-tyrosine-phosphatase (PTEN2A) are significantly regulated. The relationship between PTEN and PI(4,5)P2 is a mutually regulating mechanism, with PTEN regulating the intracellular level of PI(4,5)P2 by dephosphorylating it, and PI(4,5)P2 enhancing the activity of PTEN (Iijima et al. 2004; Pribat et al. 2011; Yoshioka et al. 2020). In rice, PI(4,5)P2 is rapidly recruited to the infection site, enveloping the invasive hyphal tip, and subsequently accumulates in the biotrophic interfacial complex (BIC) and extra-invasive hyphal membrane (EIHM) structures, playing an important role in rice-*Magnaporthe oryzae* interaction (Sha et al. 2023). Linoleic acid is an important unsaturated fatty acid. To cope with biotic stress, plants produce a series of highly modified fatty acids that play a crucial role in the plant-pathogen defense process (Pathogen-responsive gene cluster for highly modified fatty acids in tomato) (Jeon et al. 2020). In this study, in a incompatible interaction system between *Pst* and wheat, it was observed that the linoleic acid pathway was significantly enriched 18 hours after inoculation. The pathway primarily involves the lipoxygenase gene *LOX*, which in plants can convert linoleic acid and linolenic acid into hydroperoxides (Feussner and Wasternack 2002). Through a series of oxidation reactions, the hydroperoxides eventually generate jasmonic acid (JA), which participates in the JA pathway within the plant. The metabolism of linoleic acid and α-linolenic acid pathways in rice is likely to be induced by flg22 (Tang et al., 2021), which may be related with PTI response. Here, this LOX is induced in the incompatible interaction but not compatible interaction, which suggesting it may be related with ETI response. The role of LOX in wheat resistance to *Pst* is worth further investigation.

Functional annotations using eggNOG revealed the roles of DAPs in several categories, including post-translational modification, protein turnover, chaperones, translation, ribosomal structure, biogenesis, energy production, conversion, carbohydrate transport, metabolism, and signal transduction mechanisms. This suggests the complexity and coordination of multiple cellular processes involved in the defense response against *Pst*. Protein turnover denotes the equilibrium between the protein synthesis and protein degradation. In the incompatible interaction system between wheat and *Pst*, we found an enrichment of ubiquitin and its related proteins, indicating that ubiquitination-mediated protein degradation plays a crucial role in the turnover of immune proteins through rapid alteration of protein levels. Ubiquitin itself can be phosphorylated on almost every serine, threonine, and tyrosine residue (Swaney et al. 2013). However, the impact of phosphorylation modification on ubiquitin function is largely unknown. Increasing evidence suggests that phosphorylation functions upstream of ubiquitination in regulating plant innate immunity (Lu et al. 2011; Swaney et al. 2015). The findings indicate that phosphorylated ubiquitin serves as a critical mechanism for precisely modulating immune responses in plants.

By statistically analyzing the distribution of amino acid sequences before and after phosphorylation sites in all samples, the study aims to analyze the distribution patterns of amino acid sequences, specifically the conserved motifs, within phosphorylation site regions. Such analysis can uncover sequence features of modified sites and provide valuable clues for kinases and their corresponding specific substrates. In this study, Motif-x analysis showed that CYR23- and CYR31-related phosphopeptides were enriched in [xxxpSPxxx] motifs. The [xxxpSPxxx] motif is the SP-type phosphorylation sites and has been commonly reported, target for MPK (Ichimaru et al. 2022), SnRK2 (Wang et al. 2018), RLK (DeFalco et al. 2022), AGC (Hirt et al. 2014), CDK (Qi and Zhang 2020) and SLK (Stampfl et al. 2016), which are predicted phosphorylation sites for kinases involved in stress responses (Yang et al. 2022). This implies the activation of signaling pathways associated with stress and defense. The motif [xxxxSSxxxx] represents a newly identified motif where phosphorylated peptide segments related to CYR23 are enriched. Consecutive serine motif, a rarely reported motif consecutive serine motifs [xxxxSSxxxx] with relatively high occurrence was also identified by Motif-X, however, their upstream protein kinases are still unknown (Tian et al. 2014). The identification of these phosphorylation sites provides important clues for understanding the regulatory mechanisms underlying *Pst* resistance in wheat.

Further analysis of the DAPs’ interaction network could provide valuable insights into the complex molecular interactions between wheat and *Pst*. Understanding these interactions at a systems level will facilitate the identification of key regulatory nodes and potential targets for improving resistance against stripe rust in wheat.

Overall, this study elucidated the phosphoproteomic changes and molecular mechanisms underlying wheat resistance to *Pst*. These findings will contribute to our understanding of plant-pathogen interactions and provide a basis for future research aiming to enhance stripe rust resistance in wheat through the functional characterization of candidate proteins. The knowledge gained from this study can also effectively aid in the development of effective strategies for managing stripe rust in wheat crops, ensuring sustainable agriculture.

## Conclusion

In summary, our work shows that protein phosphorylation has a significant role to play in the resistance of wheat to *Pst*. Our study, which involved the use of quantitative phosphoproteomics, discovered a huge number of differentially accumulated phosphoproteins (DAPs) in the early stages of infection in both compatible and incompatible wheat-*Pst* interactions. Furthermore, our research revealed that specific phosphorylation processes were distinctly enriched in incompatible interactions, and importantly we identified changes in the phosphorylation levels of key chloroplast proteins, suggesting that photosynthesis regulation is a factor in wheat-*Pst* interaction. This was reinforced by our protein-protein interaction network analysis. Ultimately, the identification of a novel phosphorylation motif in incompatible interaction and understanding of early phosphorylation events in wheat resistance against *Pst*, opens up potential avenues for future research and strategies to combat wheat stripe rust.

## Materials and methods

### Plant materials and growth conditions

Wheat cultivar Fielder was used in this study. Fielder’s pre-germinated seeds were sown in plastic pots and transferred to a controlled climate chamber subsequently. The chamber was set at a temperature of 16 °C with a 16 hour photoperiod.

### *Pst* inoculation and sample collection

Wheat seedlings were grown and cultured for 4 weeks until they reached the two-leaf stage prior to inoculation with *Pst*. *Pst* race CYR31 (virulent to Fielder) and CYR23 (avirulent to Fielder) were utilized. Wheat cultivar Fielder that is inoculated with *Pst* race CYR31 represent the compatible interaction, and Fielder plants that were inoculated with *Pst* race CYR23 represent the incompatible interaction. Plants that were inoculated with sterilized water were used as the controls. The inoculation procedure for *Pst* followed the method described by Kang (Kang and Li 1984). Inoculated plants were subjected to 24 hours of dark and humid conditions at a temperature of 14 °C. Wheat leaves were collected at 6, 12, 18, and 24 hpi, 0.5 g per sample was collected. The collected wheat leaves were flash-frozen and stored at − 80 °C for subsequent experiments.

### Protein extraction

The sample tissue was ground into fine powder after freezing in liquid nitrogen, following which 1 mL of RIPA Lysis Buffer (Beyotime Biotechnology, Shanghai, China, Cat: P0013B) was incorporated. The effective components of the lysis buffer included 1% Triton X-100, 1% deoxycholate, and 0.1% SDS. The solution was transferred to a pre-chilled 2 mL centrifuge tube and subjected to ultrasonic treatment for 3 times, with 30 s for each time. The supernatant was gathered after centrifugation for 10 min, followed by protein precipitation using a methanol-chloroform solution. The protein that was precipitated was reconstituted in a buffer made up of Triethylammonium bicarbonate (TEAB, Sigma) which contained 8 M urea. The concentration of this protein was then assessed through the use of the BCA assay (Beyotime Biotechnology, Shanghai, China). Each sample was taken at 1 mg and added to a final concentration of 5 mM DTT. The lysate was supplemented with Dithiothreitol (DTT) (sigma, USA) at a final concentration of 5 mM, and then incubated at a temperature of 37 °C for a duration of 30 min, followed by incubation with Iodoacetamide Alkyne (IAA) (Thermo Fisher Scientific, USA) in the dark at 25 °C for 30 min to alkylate free sulfhydryl groups. The lysate underwent dilution to achieve a urea concentration of 1.5 M using 50 mM TEAB, followed by digestion with trypsin employing an enzyme/protein weight ratio of 1:100. The process was carried out overnight at a temperature of 37 °C. The peptides, post-digestion, were acid-treated with 10% Trifluoroacetic acid (TFA) (Sigma, USA), to obtain a pH of 3.0 and subsequently desalted via a Sep-Pak C18 solid-phase extraction (SPE) column (Waters Corporation, USA). Elution of the peptides from the SPE column was achieved utilizing 0.1% TFA in 80% acetonitrile followed by a thorough drying process using a vacuum centrifugal concentrator. The dried peptides were then preserved at a temperature of − 20 °C for later use.

### Phosphopeptide enrichment

The chromatography column was washed twice with 200 μL of wash buffer to balance it, and then sealed with a stopper. The lyophilized peptide sample was resuspended in 200 μL of the wash buffer and added to the balanced chromatography column. The mixture was gently mixed every 10 min and incubated for 30 min. The mixture was spun at 1000 g for 30 s to remove the flow-through, and then washed with 200 μL Binding/Wash Buffer for three times and resuspended in 200 μL of LC-MS grade water. The bound peptides were eluted in 100 μL Elution Buffer twice and immediately dried in a high-speed vacuum concentrator. The dried peptide was re-dissolved in 70 μL of 0.1% FA, and its concentration was determined using the Pierce™ Quantitative Colorimetric Peptide Assay Kit (Thermo Fisher Scientific, USA).

### Mass spectrometry analysis

The phosphopeptides were dissolved in 10 μL of 0.25% formic acid (FA) and injected into an Easy-nLC 1000 (Thermo Fisher Scientific) for peptide separation on a 45 cm analytical column (360 μm OD × 75 μm ID) packed in-house with C18 resin (2.2 μm, 100 A, Michrom Bioresources). The mobile phase buffer consisted of 0.1% FA in ultrapure water (buffer A) and 0.1% FA elution buffer (buffer B) in 80% ACN, running at a flow rate of 250 nL/min with a linear gradient of buffer B from 6% to 30% within 60 min. Easy-nLC 1000 was coupled online with Velos LTQ-Orbitrap mass spectrometer (Thermo Fisher Scientific), operated in data-dependent mode with full scan MS (resolving power of 60,000 at m/z 400 and scanning range of m/z 350–1500), followed by MS/MS collision-induced dissociation (CID) of the top 10 most abundant ions, dynamic exclusion of 60 s, and exclusion list of 500. The normalized collision energy for CID was set at 35% with an activation time of 10 ms.

### Phosphoproteomic data analysis

The wheat database (IWGSC RefSeqv1.1, 133,346 sequences) was searched utilizing Proteome Discoverer 2.4 (Thermo Fisher Scientific) and MaxQuant (Tyanova et al. 2016) to identify phosphopeptides with phosphorylated residues of serine, threonine, or tyrosine (+ 79.996 Da). This was done with a precursor mass tolerance of 15 ppm and MS/MS tolerance of 0.6 Da. Database analysis allowed for up to two missed cleavages during total trypsin digestion. The error discovery rates were set at a loose level of 0.05 and a strict level of 0.01. PhosphoRS (Taus et al. 2011) was applied for the localization of phosphorylation sites. Potential homologous proteins and different transcript products in the wheat protein library were not strictly screened during differential protein quantification. Label-free quantification was performed using Proteome Discoverer 2.4. LAXIC (Library-Assisted Extracted Ion Chromatogram) strategy, was used for quantification of phosphopeptides identified by SEQUEST with statistically significant thresholds. Relative abundance ion intensity of each peptide in different samples was normalized to the ion intensity of 160 peptides that were equally spiked in. Phosphopeptides with two significant fold-change differences (> 2 or < 0.5) between different samples in three biological replicates were considered to have different phosphorylation levels.

### VIGS in wheat

To achieve transient silencing of the *TaNDPK1* gene in Fielder, the barley stripe mosaic virus mediated virus-induced gene silencing system (VIGS) was utilized (Scofield and Brandt 2012). Specific primers were designed based on the *TaNDPK1* sequence cloned from Fielder (Table S5). The amplified fragment was cloned into the BSMV vector γ and transcribed in vitro. The transcript was then inoculated onto the two-leaf-one-shot stage wheat leaves by friction, and the infected leaves were placed in a dark and humid environment at 25–30 °C for 2 days. Afterward, the plants were exposed to light and continued to be cultivated at 25–30 °C for approximately 10 days. The leaves were monitored for the formation of viral spots, and Phytoene desaturase (*PDS*) silenced plants exhibited a striped bleaching phenotype. Wheat leaves with marked regions indicating the inoculation sites with the *Pst* races CYR23 and CYR31 were collected at 24 and 48 hpi for RNA samples and histological samples. Biomass samples (ratio of stripe rust to wheat genome) were taken 7 dpi, and phenotypes were recorded by taking photographs at 14 dpi. The observation of H_2_O_2_ detection assay were performed as previously described (Xu et al. 2020). We observed the H_2_O_2_ at 24 and 48 hpi.

### Data analysis

We used the Motif-x software (http://motif-x.med.harvard.edu/niotif-x.html) for conducting predictive analysis on the notably enhanced phosphorylation motifs in the phosphorylated peptide sections identified by mass spectrometry. The set parameters included a peptide length of 13, an incidence of 20, and a *P-*value < 10^−10^. The Protein Center of Proteome Discoverer 2.4 (Thermo Scientific) was employed to extract KEGG (Kyoto Encyclopedia of Genes and Genomes) and clusters of orthologous groups (COG) annotations for proteins. The enrichment analysis for KEGG and COG terms was carried out using DAVID (Database for Annotation, Visualization and Integrated Discovery).

### Supplementary Information

**Additional file 1.** Figure S1. KEGG analysis was performed on the DAPs at various time points during the infection. Among them, (A-D) represent the DAPs enriched in incompatible interaction at 6 h, 12 h, 18 h, and 24 h respectively; (E-H) represent the DAPs enriched in compatible interaction at 6 h, 12 h, 18 h, and 24 h respectively

**Additional file 2.** Supplementary table 1. The gene ID list of DAPs identified in wheat-Pst interaction, as well as the peptides detected at each time point

**Additional file 3.** Supplementary table 2. The result list of KEGG functional clustering analysis

**Additional file 4.** Supplementary table 3. Subcellular localization determined by CropGF for CYR23- and CYR31-related DAPs

**Additional file 5.** Supplementary table 4. The result list of COG clustering analysis

**Additional file 6.** Supplementary table 5. The primer used for VIGS-mediated silencing of the TaNDPK1 gene

## Data Availability

All data and materials are available in the paper and online supplemental files.
