# Supplementary material for: Quantitative phosphoproteomics reveals molecular pathway network in wheat resistance to stripe rust
Source: Stress Biol. 2024 Jul 1;4(1):32. doi: 10.1007/s44154-024-00170-0 (PMC11214938; doi:10.1007/s44154-024-00170-0)
Supplement: Supplementary file 1 — Additional file 1. Figure S1. KEGG analysis was performed on the DAPs at various time points during the infection. Among them, (A-D) represent the DAPs enriched in incompatible interaction at 6 h, 12 h, 18 h, and 24 h respectively; (E-H) represent the DAPs enriched in compatible interaction at 6 h, 12 h, 18 h, and 24 h respectively [file 44154_2024_170_MOESM1_ESM.docx]

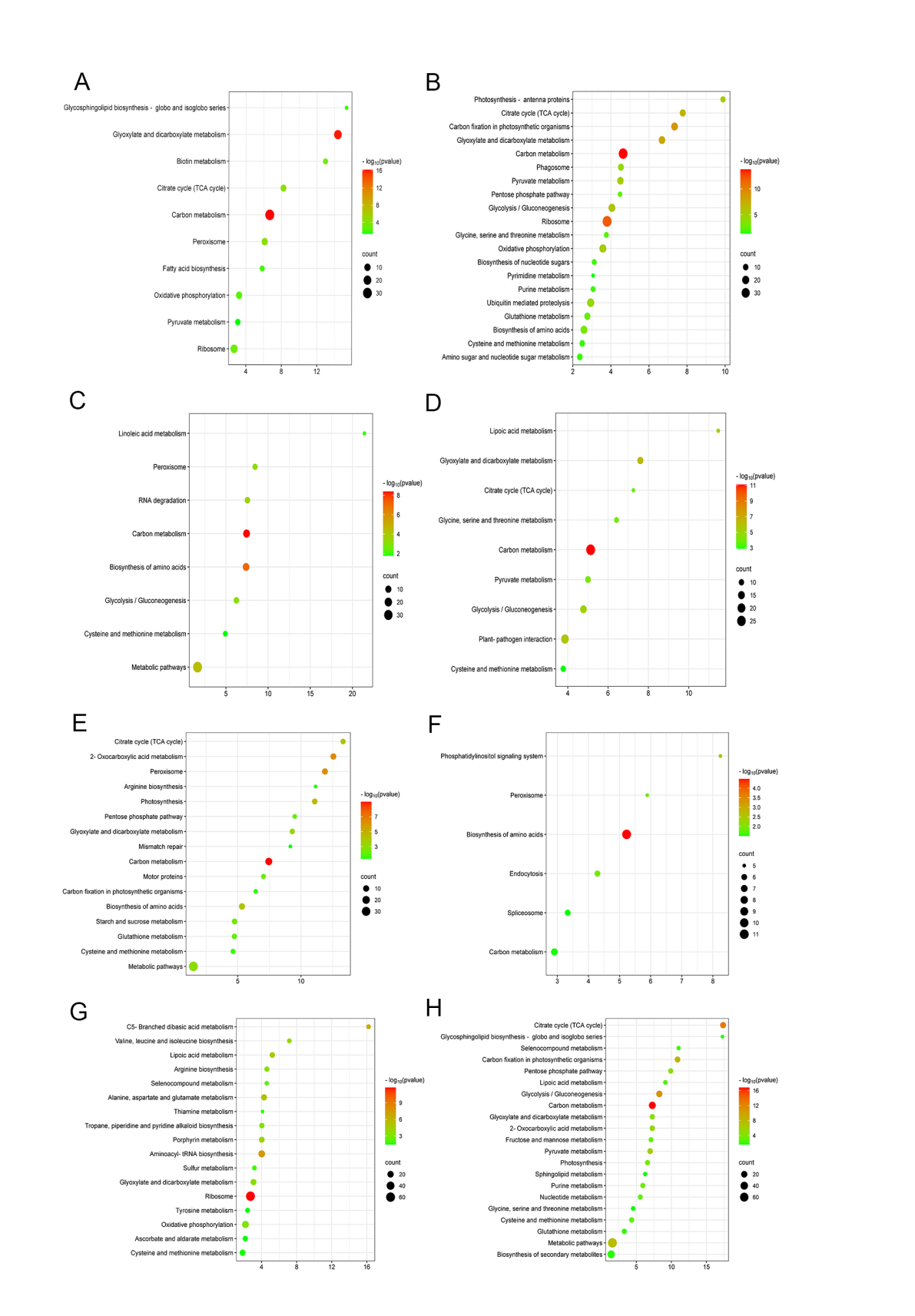


**Figure S1.** KEGG analysis was performed on the DAPs at various time points during the infection. Among them, (A-D) represent the DAPs enriched in incompatible interaction at 6h, 12h, 18h, and 24h respectively; (E-H) represent the DAPs enriched in compatible interaction at 6h, 12h, 18h, and 24h respectively.
